# Nebulized Antibiotics for Preventing and Treating Gram-Negative Respiratory Infections in Critically Ill Patients: An Overview of Reviews

**DOI:** 10.3390/antibiotics14040370

**Published:** 2025-04-02

**Authors:** Marios Karvouniaris, Despoina Koulenti, Konstantinos I. Bougioukas, Eirini Pagkalidou, Elizabeth Paramythiotou, Anna-Bettina Haidich

**Affiliations:** 1 Intensive Care Unit, AHEPA University Hospital, 54636 Thessaloniki, Greece; karvmarevg@hotmail.com; 2 Department of Critical Care, King’s College Hospital NHS Foundation Trust, SE5 9RS London, UK; 3 University of Queensland Centre for Clinical Research (UQCCR), Faculty of Medicine, The University of Queensland, 4072 Brisbane, Australia; 4 School of Medicine, Faculty of Health Sciences, Aristotle University of Thessaloniki, 54124 Thessaloniki, Greece; mpougioukas@auth.gr; 5 Department of Hygiene and Epidemiology, University of Ioannina School of Medicine, 54110 Ioannina, Greece; e.pagkalidou@uoi.gr; 6 Department of Hygiene, Social-Preventive Medicine & Medical Statistics, School of Medicine, Faculty of Health Sciences, Aristotle University of Thessaloniki, 54124 Thessaloniki, Greece; haidich@auth.gr; 7 Critical Care Department, Laikon General Hospital, 11527 Athens, Greece; eparamythiotou@laiko.gr

**Keywords:** nebulized antimicrobials, Gram-negative bacteria, ventilator-associated pneumonia, ventilator-associated tracheobronchitis, amikacin, colistin, bronchospasm

## Abstract

Background. Ventilator-associated tracheobronchitis (VAT) and pneumonia (VAP) are the most frequent nosocomial infections in the critical care setting and are associated with increased morbidity. At the same time, VAP is also associated with attributable mortality, especially when caused by difficult-to-treat (DTR) Gram-negative bacteria (GNB) that have limited treatment options. Studies have assessed the impact of nebulized aminoglycosides or colistin to improve VAT and VAP outcomes or as an adjunct to intravenous antimicrobial treatment or as a preventive approach. Objective. This overview aimed to assess systematic reviews that examine the efficacy and safety of antimicrobial nebulization for preventing and treating ventilator-associated infections in the critically ill. Methods. Systematic reviews, meta-analyses, and original randomized controlled trials and prospective observational studies were included. Searches were conducted in MEDLINE (via PubMed), the Cochrane, Epistemonikos, and PROSPERO. The methodological quality assessment was performed using standardized tools. Results. Regarding VAP treatment, the included systematic reviews presented critically low quality. The clinical response effect size to amikacin and colistin nebulization were RR 1.23 (95% CI 1.13–1.34), I^2^ = 47% and OR 1.39 (0.87–2.20), I^2^ = 56%. The main safety concern was bronchospasm with RR 2.55 (1.40–4.66), I^2^ = 0% and OR 5.19 (1.05–25.52), I^2^ = 0%. The certainty of evidence was usually very low. For VAT treatment, limited evidence showed a better clinical response and less emergence of resistant bacteria. Regarding VAP prevention, data are limited to two trials; however, only the larger one presented a low risk of bias and resulted in a reduced VAP rate. Conclusions. Delivery via nebulization might be considered in addition to IV antimicrobial treatment of GNB ventilator-associated infections. The available evidence is weak, and more studies focused on infections due to DTR-GNBs should be prioritized.

## 1. Introduction

### Background

Ventilator-associated respiratory tract infections (VARIs), i.e., ventilator-associated tracheobronchitis (VAT) and ventilator-associated pneumonia (VAP), are the most common nosocomial infections in the intensive care units (ICUs) [1]. They both occur following 48 h of invasive mechanical ventilation; VAP is infection of the lung parenchyma, while VAT is lower respiratory tract infection with no radiological infiltrate [2,3,4,5,6].

The reported VARIs incidence presents variability depending on the diagnostic criteria and the setting [1]. A multicenter observational study on the incidence of VAT provided a similar rate for VAT and VAP of 11% and 12%, respectively [5]. Aspiration (mostly micro-) of oropharyngeal secretions pooling above the endotracheal cuff and the biofilm of the artificial airway constitute the primary risk factors for the development of VARI [1].

Particularly, VAP can be problematic and difficult to treat [2,7]. Both VAT and VAP prolong the duration of mechanical ventilation and the ICU and hospital stay, while VAP presents a crude hospital mortality from 17% to as high as 42% [1]. VAP has an attributable (or excess) mortality of 10%, although it varies significantly in different reports (different case mix, different severity of illness, etc.), and can be much higher in cases of VAP with multidrug-resistant pathogens [8,9]. VAT has not been correlated with increased mortality [3].

Meanwhile, a prolonged ICU stay of about 11.5 days was positively correlated with multidrug-resistant infection development [10]. Over the last decades, several Gram-negative bacterial species (GNBs)—such as *Acinetobacter baumannii*; *Pseudomonas aeruginosa*; and members of the *Enterobacterales* family, particularly *Klebsiella pneumoniae*—have become extensively resistant (XDR) or even pandrug resistant (PDR) to antimicrobials [11]. In addition, if the bacterial isolates are resistant to all beta-lactams and the fluoroquinolones, difficult-to-treat resistant (DTR), they are associated with higher overall mortality compared to less resistant phenotypes [12]. Regrettably, this overwhelming resistance to many currently available drugs goes along with a few efficacious novel antimicrobials already approved or pending approval [13,14].

Apart from intravenous (IV) antimicrobial administration, another treatment option for VAP and VAT is direct delivery, via nebulization, of the currently available antimicrobials to the infected organ, i.e., the tracheobronchial tree and the lung parenchyma. The present guidelines, published by the Infectious Diseases Society of America, for the treatment of VAP recommend nebulized adjunct to the IV-administered antimicrobials for those highly resistant GNBs. The mechanically ventilated patients in the ICU often receive nebulized (aerolized) antimicrobials as part of a treatment regimen for respiratory infections, despite the limited evidence about their efficacy [7,15,16].

The antimicrobials usually used for nebulization, particularly, for the XDR-GNBs, are the polymyxins, polymyxin B and colistin (polymyxin E), and the aminoglycosides, amikacin gentamycin and tobramycin. Historically, they have been used for decades in the management of chronic respiratory infections [17,18]. Regarding acute ventilator-associated infections, two different strategies are suggested for this treatment modality: (a) adjunctive, meaning that the same antimicrobial, which is already administered through the IV route, is also added on via the airways by nebulization, and (b) substitution, meaning that the agents are delivered by nebulization, instead of the intravenous route, and added to other first-line IV antimicrobials [15].

Meanwhile, the nebulization mode varies between studies. The aerosol generator type matters, as it decides the size of the antimicrobial particles. Indeed, a size of <5 μL enables the delivery of the drug into the distal airways and the lung parenchyma, the locus of the infection, as opposed to larger particles, which are deposited in the ventilator circuit and the proximal airways [15,19]. Three types of nebulizers are available: jet, ultrasonic, and vibrating mesh, with the last considered the most efficient. However, there are many prerequisites for avoiding deposition of the antimicrobial agents in the respiratory circuit and the proximal airways: patient–ventilator coordination via brief sedation and specific circuit changes and specific ventilator settings during nebulization [19]. Pharmacokinetic studies have examined the impact of the delivered dose on the plasma and epithelial lining fluid levels [20,21,22]. These levels vary between different assessments; thus, the authors suggested an increase in the nebulized dose [20,21,23]. Nonetheless, the antibiotic levels in the epithelial lining fluid are many-fold higher than those of plasma [20,21,22]. Antimicrobial administration via nebulization can boost its pharmacokinetics, i.e., increase drug delivery to the focus of infection without increasing the total daily dose of the IV prescription. Of note, this local delivery may decrease the duration of IV treatment and may also decrease the systemic antimicrobial toxicity (need for lower IV doses and avoiding toxic drug plasma concentrations) [24,25].

The pertinent literature lacks large randomized controlled trials, while the position paper of the European Society of Clinical Microbiology and Infectious Diseases suggests avoiding antibiotic nebulization during invasive mechanical ventilation in standard clinical practice due to weak evidence of efficacy and safety concerns [15]. Despite the above, this position paper was published seven years ago, and an updated literature evaluation that summarizes the available evidence from systematic reviews, in the form of an overview, is lacking [26,27,28]. In this report, we aimed to collect and summarize data of related systematic reviews and meta-analyses on the efficacy and safety of nebulized antimicrobials for preventing or treating Gram-negative respiratory infections in the ventilated critically ill population. Regarding treatment, we focus on comparing IV-only antimicrobial administration versus antibiotic nebulization adjunct to the IV delivery. Finally, we complement our evaluation of the topic with the corresponding confidence across all available outcomes.

## 2. Results

**Figure 1:**
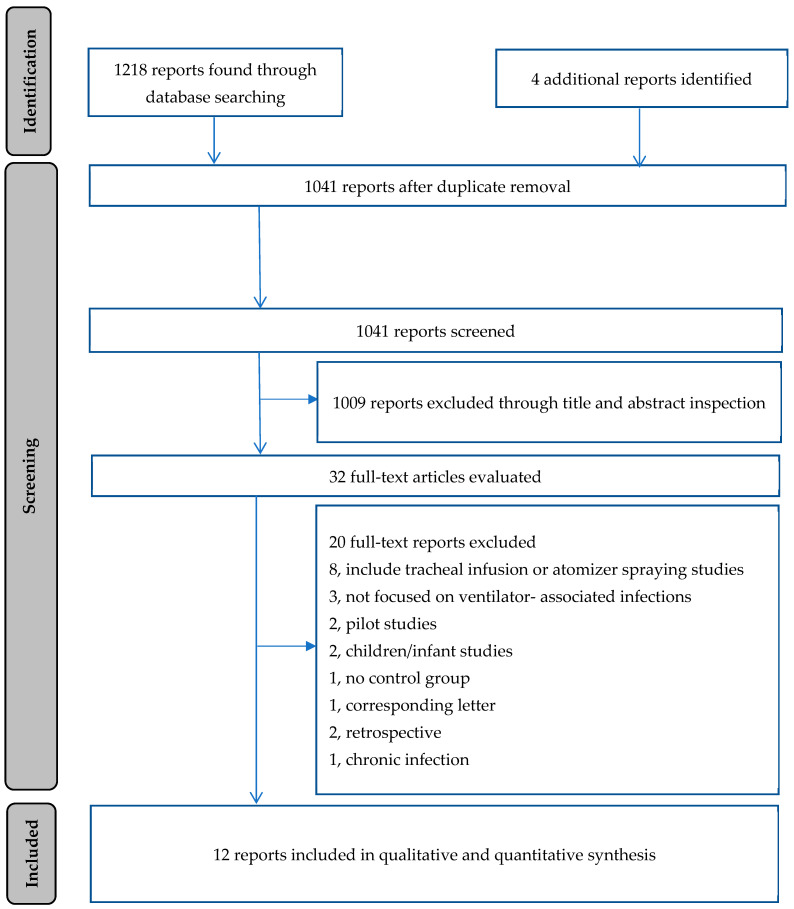
PRISMA flow diagram.

The flow chart (Figure 1) presents the detailed screening and selection steps; please see the Supplementary Material for the search strategy. Initially, 1222 records were identified. Following the removal of duplicates and the title and abstract screening, 32 full-text reports were finally selected for further evaluation. Finally, the overview synthesis included nine meta-analyses [29,30,31,32,33,34,35,36,37], one systematic review [38], and two primary studies [39,40].

Twenty full-text reports were excluded for various reasons and are presented in Table S1 [41,42,43,44,45,46,47,48,49,50,51,52,53,54,55,56,57,58,59,60].

### 2.1. Prevention Assessment

The existing meta-analyses included studies with antibiotic instillation or spraying into the trachea and, thus, were excluded (Table S1). There were two available primary studies, both randomized controlled studies, comparing nebulized drugs to nebulized saline placebos (Table 1). The first was a single-center study of nebulized colistin, 500,000 units thrice daily, vs. saline placebo, taken for ten days or until extubation. It included 168 patients and did not result in less 30-day VAP. However, it resulted in less VAP due to Gram-negative pathogens [39]. The second study was a recent large multicenter trial that used once-daily nebulized amikacin, at a dose of 20 mg/kg of ideal body weight, or saline for a maximum of three days. The intervention resulted in a lower 28-day VAP rate [40]. Contrary to the former study’s use of a jet nebulizer, the latter study’s antimicrobial was nebulized with a vibrating mesh device, which is more sophisticated and technologically advanced. Meanwhile, the former study raises some concerns for performance and detection bias, as an open-label investigation, in contrast to the latter that presented a low risk of bias.

Apart from VAP, the VAT rate was reported in the aforementioned single-center study; however, that rate did not differ between the investigational and the control group [39].

### 2.2. Treatment Assessment

Evaluation of antibiotic nebulizer treatment was performed with ten systematic reviews (nine of them were meta-analyses) [29,30,31,32,33,34,35,36,37,38]. The report characteristics are displayed in Table 1.

The R code for the overlap evaluation and the AMSTAR-2 assessment, with the pertinent visualizations, is displayed in the Supplementary Materials.

### 2.3. Methodological Quality Evaluation

The overall methodological status of the included systematic reviews and meta-analyses was evaluated with the AMSTAR-2 tool. The quality of the systematic reviews/meta-analyses was appraised as critically low in all the reviews (Table 1 and Table S2 and Figures S1 and S2). No registered protocol and lack of the excluded full-text report list were the commonly encountered critical domain flaws.

### 2.4. Outcomes—Effects of Interventions

A summary of the effect sizes, the heterogeneity I^2^ statistic, for all efficacy and safety outcomes is displayed in Table 2, while the corresponding corrected covered area index (CCA) and the pertinent, for every outcome, number of reviews are presented in Table 3.

### 2.5. Clinical Response

The clinical response to nebulized antibiotic administration was evaluated by nine reviews [29,30,31,32,33,34,36,37], while the CCA was 15.4% (Supplementary Material, Figure S3). The Sole-Lleonart report contained only four studies [34]. The reviews that provided the effect sizes were the Qin and Zhang reports, which did not present any overlap, as they investigate different drugs, amikacin and colistin, respectively [30,35]. The corresponding respective effect size measures were RR 1.23 (95% CI 1.13–1.34), I^2^ = 47%, and OR 1.39 (0.87–2.20), I^2^ = 56%.

### 2.6. Bacterial Response and Eradication

Bacterial eradication was evaluated in seven reviews [29,30,31,32,33,35,37]. The CCA was 16.7% (Figure S4). The selected review was the Sella report, with OR 2.63 (1.36–5.09), I^2^ = 77%.

### 2.7. Mortality

Overall (all-cause) mortality was assessed by eight reviews [30,31,32,33,34,35,36,37]. The study overlap between the reviews was 18.4% (Figure S5). The reports used for effect size extraction were the Qin and Vardakas ones; the respective amikacin and colistin effects were non-significant at RR 1.17 (0.98–1.50), I^2^ = 0%, and RR 0.94 (0.81–1.08), I^2^ = 16% [35,36].

Regarding pneumonia-associated mortality, three reviews used it as an outcome [32,35,36], with a CCA of 9.1% (Figure S6). The Qin and Valachis reports were the most comprehensive [31,35]. Moreover, they were complementary and non-overlapping. The corresponding respective effect sizes of amikacin and colistin were RR 1.12 (0.82–1.52), I^2^ = 0%, and OR 0.58 (0.34–0.96), I^2^ = 46%.

### 2.8. Other Clinical Outcomes

The effect of nebulization treatment on the duration of mechanical ventilation was evaluated by three reviews [32,34,35], with a CCA index of 10% (Figure S7). Regarding the most comprehensive, the Zampieri and Qin reviews, the effect of the studies was non-significant with high levels of heterogeneity.

The ICU length of stay was also evaluated by two reports [32,35]. The available Zampieri and Qin reviews did not overlap (Figure S8); however, the effect size was not significant and presented high heterogeneity.

### 2.9. VAT Treatment

Antimicrobial nebulization did not improve mortality or the mechanical ventilation duration, although it decreased the clinical pulmonary infection score by a mean difference of 3.11 (95% CI −0.04 to −6.18, I^2^ = 90%) in two small trials. Of interest, the intervention decreased the emergence of resistant strains compared with the controls (RR 0.18, 95% CI 0.05 to 0.64, I^2^ = 0) [34].

### 2.10. Adverse Effects

The evaluated adverse effects profile, concerning nebulized adjunct antibiotics, included nephrotoxicity, bronchospasm, and overall cardiorespiratory complications.

Regarding nephrotoxicity, it was assessed in seven reviews [29,31,32,33,34,35,37], with a CCA of 15.9% (Figure S9). The selected Qin and Liu reviews evaluated the renal failure rate with a respective effect size of amikacin and colistin of RR 0.82 (0.60–1.12), I^2^ = 2%, and OR 1.11 (0.69–1.80), I^2^ = 23% [33,35].

Meanwhile, bronchospasm was evaluated in two non-overlapping reviews, by Qin and Zhang (Figure S10) [30,35]. The reviews provided the effect sizes RR 2.55 (1.40–4.66), I^2^ = 0%, and OR 5.19 (1.05–25.52), I^2^ = 0%.

The cardiorespiratory complications, which include not only bronchospasm but also hypoxemia, obstruction of the expiratory filter, arrhythmias, and cardiorespiratory arrest, were estimated to occur similarly between the control and the experimental group (risk difference equals zero, 95% CI from −0.04 to 0.04), I^2^ = 65% [34].

### 2.11. Certainty of Evidence

The Qin and Zhang reports directly provided the GRADE evidence profile for the outcomes in the meta-analyses, regarding amikacin and colistin, respectively [30,35]. Regarding clinical response, they presented a moderate risk of bias and inconsistency. The imprecision was serious due to the small sample size of most reviewed studies. The respective quality of the evidence was moderate and low to very low.

Concerning all-cause mortality, they displayed a moderate risk of bias, limited inconsistency, and moderate imprecision. Meanwhile, a small-study effects bias was detected in the Zhang review. The corresponding quality of the evidence was moderate and low to very low.

The certainty of evidence for pertinent efficacy and safety outcomes is summarized in Table 4. The evidence quality was, at best, moderate and often very low to low. The certainty of evidence on amikacin adjunct nebulization therapy vs. the control group was better compared to that of colistin treatment vs. the control group.

The significant effects of the intervention are limited to microbiological eradication and the adverse effect of bronchospasm. Regarding the clinical response outcome, it is affected by amikacin, whereas the effect of colistin was insignificant.

## 3. Discussion

The present report overviews the literature regarding the comparison between antimicrobial nebulization plus IV antimicrobials compared to the sole IV antimicrobial administration for the treatment of GNB-VARIs. The overall evidence for the efficacy of this treatment modality is weak to very weak, regarding multiple outcomes, including the clinical response to treatment or mortality or microbiological eradication, while nebulization is relatively safe. Meanwhile, VAT treatment efficacy data is scarce. In respect to a particular drug, evidence for therapeutic efficacy is low for amikacin and very low for colistin. Finally, evidence for the overall VAP prevention via nebulization is scarce.

Adjunct nebulization with aminoglycosides and polymyxins did not impact mortality. Although mortality is an overall straightforward endpoint in the ICU, many factors can neutralize treatment effects [61]. Clinical and bacteriological response may be better coupled to the desired treatment effect. Compared to placebo, amikacin presented a better clinical response than colistin; however, a direct comparison was not available in the literature. Bacterial eradication, in turn, was evident following nebulization with either drug, although cultured secretions may have been falsely negative because of the presence of antibiotics [62]. Very low evidence shows that the intervention did not affect the ICU length of stay and mechanical ventilation duration. Regarding adverse events, it did not have an impact on renal function (no increased nephrotoxicity with adjuvant nebulized antibiotics), possibly due to low systemic concentrations; however, some patients presented bronchospasm, a complication that might be prevented with improved airway humidification and, perhaps, pretreatment with beta2 agonists [24,63]. Based on these findings, it can be deducted that this adjuvant treatment modality could be considered for the management of XDR-GNB-VAP. Regarding VAT management, limited data suggest nebulization might reduce antimicrobial resistance development [34].

This study’s findings align with the current recommendations on nebulized aminoglycosides and colistin, which question their clinical value for the routine treatment of GNB-VARIs [7,15]. The position paper of the European Society of Clinical Microbiology and Infectious Diseases suggests avoiding the intervention, regardless of the pathogen susceptibility status, while the Infectious Diseases Society of America guidelines suggest adding nebulization to the IV-delivered aminoglycosides and polymyxins for treating VAP due to GNB that are only susceptible to those drugs [7,15].

The limited evidence on antimicrobial delivery via nebulization is magnified by the variable dosing schemes, either different doses or dosing intervals, and different devices for delivery of the drug to the lungs (Table 1). The pharmacokinetics of nebulized antibiotics is complex, while the diluent volume, and thus the antimicrobial concentration, impacts the drug’s stability and nebulization time [64,65]. Meanwhile, an experimental study demonstrated that components of sputum, i.e., mucin, bind and inactivate most of the delivered free drug in the lungs [66]. Administering a low dose, particularly for colistin, might explain some outcome inconsistencies, as high-dose nebulized drug might improve the outcome. The sole nebulization of five million colistin units thrice daily produced a comparable clinical effect with intravenous antibiotics for treating multidrug-resistant VAP due to less resistant pathogens [67]. Following the above reference, there has been a trend for a decade favoring a higher antimicrobial, i.e., colistin, dosing to improve the drug’s delivery to the lung parenchyma [67]. Delivery often requires modern nebulizer equipment but can be hindered by inadequate practices [19,68]. However, the investigators of the topic used different types of nebulizers, while antimicrobial delivery was not always strictly protocolized (Table 2). It is a demanding procedure, where the medical and nursing staff should be thoroughly educated to optimally deliver the drug [69]. Moreover, the sophisticated vibrating mesh nebulizers were also shown to fail during experimental nebulization [70]. The above demonstrate the need for augmenting antimicrobial delivery by higher dosing and the optimal nebulization technique. Meanwhile, the therapeutic drug monitoring of nebulized antimicrobials is complex. The epithelial lining fluid levels, which are occasionally measured at present, can overestimate the interstitial concentrations of interest. What the future holds might be the invasive technique of lung microdialysis [71,72,73]. Still, the antibiotic levels following nebulization surpass those after IV delivery for the treatment of respiratory infections [20,21,22,74].

According to the above, improved colistin and aminoglycoside dosing and delivery might render this treatment modality more useful for daily ICU practice. Special cases where adjunct nebulization could be administered are immunosuppressed patients, initial IV treatment failure, or VAP relapse [75]. For MDR/XDR GNBs, and particularly for pathogens only susceptible to aminoglycosides and polymyxins, nebulized administration to the focus of infection, i.e., trachea/lungs, might be crucial. On the other hand, adjuvant nebulization might be less needed for VARI by more susceptible GNBs [7,75].

A prime concern regarding this overview is the poor methodological quality of the systematic reviews on the topic. Indeed, the evaluation of the available systematic reviews displayed low or critically low evidence for nebulization. Meanwhile, there are no large multicenter trials available to better evaluate antimicrobial nebulization for treating VAP. Most available studies included in the reviews were either small or observational (Table 1). Therefore, the evidence is biased and weak. This is, to our knowledge, the first overview about nebulized antimicrobials, thus not allowing comparison to other reports. The available weak data primarily concern nebulized antibiotics as adjunct treatment, while their use for substituting intravenous drugs remained minimally explored. Meanwhile, the best available evidence on prevention is merely a large multicenter trial of amikacin [40].

This overview has several limitations. Firstly, the reviewed meta-analyses are of small single-center RCTs and mostly of observational studies, precluding agreeable evidence quality. They are open to the small-study effect and confounding factors. Secondly, the meta-analyzed studies include a mixed population of patients with difficult-to-treat and less resistant bacteria, while dosing varied between the studies. Thirdly, it did not evaluate and excluded the available prevention meta-analyses, as they included old studies, where the authors examined the obsolete practice of delivering antimicrobials via instillation or hand atomizer spraying into the trachea.

## 4. Materials and Methods

This overview was registered at Open Science Framework, and the protocol is available at https://osf.io/g6qr9 (last accessed on 17 June 2024). We conducted this study in accordance with the Cochrane Handbook [26]. The reporting of this overview adhered to the standards outlined by the Preferred Reporting Items for Overviews of Reviews (PRIOR) statement, with the aid of a checklist [76].

### 4.1. Inclusion and Exclusion Criteria

#### 4.1.1. Inclusion Criteria

The inclusion criteria were as follows: (1) Systematic reviews and meta-analyses examining nebulized antibiotics for treatment of or prophylaxis from VARI, focusing on the aminoglycosides and the polymyxins, i.e., amikacin, gentamycin, tobramycin, colistin, and polymyxin B. (2) The reviews should clearly report efficacy or safety outcomes. (3) Only articles in English were eligible. (4) Recent original research articles could also be included if they were not part of a systematic review. Those articles could be either randomized controlled or prospective observational studies.

#### 4.1.2. Exclusion Criteria

The exclusion criteria were as follows: (1) The systematic reviews contained no comparison data between an investigational and a control group. (2) Studies that included non-ventilated patients. (3) Studies or meta-analyses in which delivery of the antibiotic in the tracheobronchial tree was performed via instillation or spraying with a hand atomizer. (4) Retrospective observational primary studies. (5) Small, primary studies including less than 50 patients, and clinical investigational or pilot studies. (6) Experimental primary studies. (7) Infant or children reviews or studies. (8) Chronic infections’ treatment.

#### 4.1.3. Definitions

Multidrug-resistant isolates are defined whenever they are resistant to at least an agent of three or more classes [11]. Extensively drug-resistant (XDR) isolates are considered when they retain susceptibility to only one or two antimicrobial classes and are not susceptible to at least a member of the rest of the drug categories [11]. Pandrug-resistant (PDR) is defined as the bacterial isolates that are not susceptible to every antimicrobial agent in all drug categories [11]. Difficult-to-treat resistant (DTR) GNB was considered as an isolate demonstrating an intermediate or resistant phenotype to all reported agents in the carbapenem, beta-lactam, and fluoroquinolone classes [12].

### 4.2. Information Sources and Search Strategy

A comprehensive search was performed in MEDLINE (via PubMed), the Cochrane Database of Systematic Reviews, Epistemonikos, and PROSPERO to identify systematic reviews and primary studies of nebulized (or aerolized) antimicrobials for preventing or treating respiratory infections in the ventilated critically ill population. The period of searching spanned from the database inception until 1 December 2024. Regarding PubMed, we used the following MeSH terms: “Nebulizers and Vaporizers”, “Administrations, Inhalation”, “Pneumonia, Ventilator-Associated”, “Anti-Bacterial Agents”, “Outcome”, and “Drug-Related Side Effects and Adverse Reactions”. Other search keywords were “aerolized”, “nebulized”, “inhaled”, “instilled”, “antibiotics”, “antimicrobials”, “ventilator-associated tracheobronchitis”, “ventilator-associated pneumonia”, “pulmonary infections”, “lung infections”, “respiratory tract infections”, “hospital-acquired pneumonia”, “bacterial eradication”, “clinical cure”, “microbiological cure”, “mortality”, “length of stay”, and “side effects”. Searches were supplemented by backward citation chasing.

### 4.3. Study Selection

The articles’ title and abstract were initially and independently screened for relevance by two authors (M.K., El.P). The full text of every initially retrieved article was later evaluated for eligibility. The final eligible articles were selected after disagreements were settled by the senior authors (A.B.H., K.I.B., D.K.).

Overlap between pairs of the systematic reviews for each outcome of interest was evaluated by calculating the corrected cover area (CCA)—formula (as a percentage) and graphically displayed by heatmaps using the “ccaR” R package [77,78,79]. In the cases of very high overlap between systematic reviews, the review with the best overall quality based on the Assessment of Methodological quality of Systematic Reviews (AMSTAR-2)—the most relevant, recent, or comprehensive review that could provide the best available effect size—was chosen [26,80].

### 4.4. Data Extraction

The following data variables were extracted: year of publication, country, patients’ number, nebulization method, purpose of nebulization (treatment or prevention), dose, and frequency of delivery. Moreover, outcome data of interest were as follows: clinical cure, microbiological cure, mortality, pneumonia-associated mortality, the adverse effect rate, mechanical ventilation duration, and ICU length of stay. Apart from the text, the Supplementary Materials of the reports were searched to avoid missing data. Two reviewers (M.K., El.P.) independently extracted data, and any discrepancies were resolved by a third reviewer (D.K.).

### 4.5. Methodological Quality and Risk of Bias Assessment

The selected reports were evaluated for quality with the 16-item AMSTAR-2 tool. Every item was answered with “yes”, “partial yes”, or “no”, and ratings on the overall confidence in systematic reviews’ results (“High”, “Moderate”, “Low”, or “Critically Low”, based on seven items–critical domains) were calculated [59]. The evaluation was graphically presented using the “amstar2Vis” R package, version 4.0.3 [81].

Whenever a relevant primary study, not yet included in a systematic review, was available for the present overview, it was assessed by two independent reviewers (M.K., Ei.P), with the appropriate tool for the type of study, either risk of bias (ROB-2) for randomized trials or the Risk of Bias in non-randomized studies of interventions (ROBINS-I) for observational studies [82,83].

### 4.6. Data Analysis

Data were analyzed by R version 4.0.3 [84]. We prioritized extracting the results, effect sizes, and heterogeneity assessment (I^2^ values) from the systematic reviews [26].

### 4.7. Certainty of Evidence Assessment

The body of available evidence regarding the intervention outcomes was assessed by two reviewers (M.K., El.P.) using the Grading of Recommendations Assessment, Development and Evaluation (GRADE) approach [85,86]. These reviewers then reached a consensus assessment on the certainty of the evidence across outcomes. Our priority was to extract the assessment of the studied reviews [26].

We finally summarized the nebulization efficacy and safety with the corresponding confidence across all available outcomes.

## 5. Conclusions

To summarize, adjunct nebulization to the intravenous antimicrobial delivery might be an option for treating Gram-negative ventilator-associated respiratory infections in the ICU. However, evidence in favor of this therapeutic modality is overall weak for the most commonly used antimicrobials, amikacin and colistin.

There is a need for more randomized controlled trials focusing exclusively on the more challenging infections caused by multidrug-resistant and extensively drug-resistant Gram-negative bacteria. Considering ventilator-associated pneumonia prevention, a favorable large amikacin trial needs to be further validated.

## Figures and Tables

**Table 1:** Studies’ characteristics.

Author, Year (Ref)	Study Type	Included Studies	Studies/Patients, N	Bacteria Commonly Treated	Nebulization Strategy	Nebulized Drug	Daily Nebulized Dose, Range	Device	Primary Outcome	AMSTAR-2
Treatment studies										
Sella, 2024 [29]	Meta-analysis	RCT	11/1472	NS	Adjunctive	Amikacin, colistin, polymyxin B, tobramycin	NS	variable	Microbiological eradication	Critically low
Zhang, 2023 [30]	Meta-analysis	RCT, observational	10/950	Ab, Kp, Pa	Adjunctive	Colistin	2–12 MU	variable	Clinical response	Critically low
Valachis, 2015 [31]	Meta-analysis	Observational, RCT *	8/724	Ab, Kp, Pa	Adjunctive/substitutive §	Colistin	2–5 MU	variable ¶	Clinical response	Critically low
Zampieri 2015 [32]	Meta-analysis	RCT, observational	12/885	Ab, Kp, Pa	Adjunctive/substitutive §	Amikacin, colistin, tobramycin	200–1200 mg 2–4 MU 200–600 mg	variable	Clinical cure	Critically low
Liu, 2015 [33]	Meta-analysis	Observational	9/672	Ab, Kp, Pa	Adjunctive/substitutive §	Colistin	2–4 MU	NS	Multiple outcomes	Critically low
Russell, 2016 [38]	Systematic review	RCT	6/305	NS	Adjunctive	Amikacin, colistin, tobramycin	NS	NS	Clinical cure	Critically low
Sole-Lleonart, 2017 [34]	Meta-analysis	RCT, observational	11/826	NS	Adjunctive/substitutive §	Amikacin, colistin, gentamycin, tobramycin	NS	variable	Multiple outcomes	Critically low
Qin, 2021 [35]	Meta-analysis	RCT	13/1733	NS	Adjunctive	Amikacin	800–1600 mg	variable	Clinical response	Critically low
Vardakas, 2018 [36]	Meta-analysis	Observational	13/1135	Ab, Kp, Pa	Adjunctive	Colistin	2–6 MU	NS	All-cause mortality	Critically low
Xu, 2018 [37]	Meta-analysis	RCT, observational	17/1367	Ab, Kp, Pa	Adjunctive/substitutive §	Amikacin, colistin, tobramycin	300–1750 mg 1–4 MU 240–600 mg	variable	Multiple outcomes	Critically low
Prevention studies †										
Karvouniaris, 2015 [39]	RCT	NA	NA/	NA	NA	Colistin	500,000 iu TDS for the first 10 ICU days	jet nebulizer	30-day VAP incidence	NA
Ehrmann, 2023 [40]	RCT	NA	NA/	NA	NA	Amikacin	20 mg/kg #, OD for 3 consecutive days	vibrating mesh nebulizer	28-day VAP incidence	NA

Abbreviations: Ab, *Acinetobacter baumannii;* AMSTAR, A MeaSurement Tool to Assess systematic Reviews; Ec, *Escherichia coli;* GN, Gram-negative; iu, international units; Kp, *Klebsiella pneumoniae;* MDR, multidrug resistant; MU, million units; N, number; NA, not applicable; NS, not specified; OD, once daily; Pa, *Pseudomonas aeruginosa;* RCT, randomized controlled trial; Ref, reference; TDS, thrice daily; VAP, ventilator-associated pneumonia. * Only one RCT was included. § Limited data. ¶ In half the studies, it was not reported. † The control was nebulized saline, while the intervention lasted until extubation. # Of ideal body weight.

**Table 2:** Summary of pooled efficacy and safety effect estimates for treating VAP with nebulized antibiotics.

Outcome	Nebulized Antibiotic	Pooled Effect Estimates (95% CI)	I^2^ Statistic
Clinical response	Amikacin	RR 1.23 (1.13–1.34)	47%
	Colistin	OR 1.39 (0.87–2.20)	56%
Bacterial eradication	Amikacin	RR 1.51 (1.35–1.69)	6%
	Colistin	OR 2.21 (1.25–3.92)	64%
All-cause mortality	Amikacin	RR 1.17 (0.98–1.50)	0%
	Colistin	RR 0.94 (0.81–1.08)	16%
Pneumonia-associated mortality	Amikacin	RR 1.12 (0.82–1.52)	0%
	Colistin	OR 0.58 (0.34–0.96)	46%
Mechanical ventilation duration, days	Amikacin	MD −0.45 (−2.69 to 1.78)	84%
	Amikacin/colistin/tobramycin	SMD −0.10 (−1.22 to 1.00)	96.5%
ICU length of stay, days	Amikacin	MD −0.31 (−2.08 to 1.45)	67%
	Amikacin/colistin/tobramycin	SMD 0.14 (−0.46 to 0.73)	89.2%
Nephrotoxicity	Amikacin	RR 0.82 (0.60–1.12)	2%
	Colistin	OR 1.11 (0.69–1.80)	23%
Bronchospasm	Amikacin	RR 2.55 (1.40–4.66)	0%
	Colistin	OR 5.19 (1.05–25.52)	0%

Abbreviations: CI, confidence interval; ICU, intensive care medicine; MD, mean difference; OR, odds ratio; RR, risk ratio; SMD, standardized mean difference; VAP, ventilator-associated pneumonia.

**Table 3:** Summary of overlap estimates between the eligible reviews for every outcome of interest.

Outcome	Number of Reviews, n	CCA Percentage, %
Clinical response	9	15.4
Bacterial eradication	7	16.7
All-cause mortality	8	18.4
Pneumonia-associated mortality	3	9.1
Mechanical ventilation duration	3	10
ICU length of stay	2	0
Nephrotoxicity	7	15.9
Bronchospasm	2	0

Abbreviations: CCA, corrected covered area; ICU, intensive care unit.

**Table 4:** Evidence summary on nebulization treatment intervention vs. control.

	Amikacin vs. Control	Colistin vs. Control
Significance Level/Certainty of Evidence		
Outcome		
Clinical response	Significant/Moderate	Non-significant/Low–very low
Microbiological eradication	Significant/Low	Significant/Low–very low
All-cause mortality	Non-significant/Moderate	Non-significant/Low–very low
Infection-associated mortality	Non-significant/Moderate	Non-significant/Very low
Mechanical ventilation duration	Non-significant/Very low	Non-significant/Very low *
Length of ICU stay	Non-significant/Low	Non-significant/Very low
Nephrotoxicity	Non-significant/Moderate	Non-significant/Very low
Bronchospasm	Significant/Moderate	Significant/Low

* The majority received colistin treatment while the rest aminoglycosides. Abbreviations: ICU, intensive care unit.

## Data Availability

No new data were created or analyzed in this study. The R code is available in the Supplementary Materials.

## Supplementary Materials

The following supporting information can be downloaded at: https://www.mdpi.com/article/10.3390/antibiotics14040370/s1, Figure S1: AMSTAR 2 barplot; Figure S2: AMSTAR 2 overall confidence; Figure S3: Clinical response. CCA percentage 15.4%; Figure S4: Microbiological eradication. CCA percentage 16.7%.; Figure S5:All-cause mortality. CCA percentage 18.4%; Figure S6:. Pneumonia-associated mortality. CCA percentage 9.1%; Figure S7: Duration of mechanical ventilation. CCA percentage 10%; Figure S8. ICU length of stay. CCA percentage 0%; Figure S9. Nephrotoxicity. CCA percentage 15.9%; Figure S10. Bronchospasm. CCA percentage 0%; Table S1. Exclusion list after full-text search; Table S2. AMSTAR 2 items.

## Abbreviations

The following abbreviations are used in this manuscript:

AMSTAR	Assessment of Methodological Quality of Systematic Reviews
CCA	Corrected Covered area
DTR	Difficult-to-treat resistant
GNB	Gram-negative bacteria
GRADE	Grading of Recommendations Assessment, Development and Evaluation
IV	Intravenous
PDR	Pandrug resistant
VAP	Ventilator-associated pneumonia
VARI	Ventilator-associated respiratory tract infections
VAT	Ventilator-associated tracheobronchitis
XDR	Extensively drug resistant
