# Supplementary material for: Nebulized Antibiotics for Preventing and Treating Gram-Negative Respiratory Infections in Critically Ill Patients: An Overview of Reviews
Source: Antibiotics (Basel). 2025 Apr 2;14(4):370. doi: 10.3390/antibiotics14040370 (PMC12024070; doi:10.3390/antibiotics14040370)
Supplement: Supplementary file 1 [file antibiotics-14-00370-s001.zip › antibiotics-3477235-supplementary.pdf]

antibiotics

SUPPLEMENTARY MATERIAL

Table S1. Exclusion list after full-text search.

Author, Journal, Year	Reason for exclusion	Main text reference
Zha, Eur J Med Res, 2023	Includes antimicrobial instillation studies	41
Povoa, J Crit Care, 2018	Includes antimicrobial instillation studies	42
Tang, J Crit Care, 2021	Includes antimicrobial instillation studies	43
Sweeny, Clin Microbiol Infect, 2019	Includes antimicrobial instillation studies	44
Lu, Heliyon, 2023	Includes children studies	45
Wu, Front Pharmacother, 2021	Not focused on ventilator-associated infections, overview	46
Honore, J Crit Care, 2024	Corresponding letter	47
Hassan, Crit Care Med, 2018	Included non-ventilated patients	48
Levchenko, Acta Biomed, 2023	Pilot study	49
Angermair, Anaesth Crit Care Pain Med, 2023	Pilot study	50
Bharati, Ann Card Anaesth, 2022	Children study	51
Tejada, Antibiotics, 2022	Chronic infection	52
Aygun, Antibiotics, 2019	Retrospective, children study	53
DePascale, Antibiotics, 2023	No control group	54
Andrianopoulos, Antibiotics, 2024	Retrospective study	55
Buendia, BMC Pulm Med, 2024	Includes antimicrobial instillation studies	56
Li, Crit Care Med, 2024	Includes antimicrobial instillation studies	57
Hsu, International J Antimicrob Agents, 2024	Includes antimicrobial instillation studies	58
Gao, PeerJ, 2024	Includes atomizer spraying studies	59
Luque Paz, Ann Intensive Care, 2024	Not focused on ventilator-associated infections	60

 https://doi.org/10.3390/antibiotics14040370

Table 2. S. AMSTAR 2 items.

								AMSTAR 2 items									
**Reviews**	**1. PICO components[A]**	**2.* Pre-established protocol[B]**	**3. Explanation of included studies' design[A]**	**4.* Comprehensive search strategy[B]**	**5. Duplicate study selection[A]**	**6. Duplicate data extraction[A]**	**7.* List of excluded studies and justification[B]**	**8. Description of included studies[B]**	**9.* Assessment of RoB in included studies[B,C]**	**10. Funding sources[A]**	**11.* Use of appropriate statistical methods[C,D]**	**12. RoB impact on synthesized results[D]**	**13.* Results interpretation with RoB reference[A]**	**14. Heterogeneity explanation[A]**	**15.* Publication/ small study bias investigation[D]**	**16. Conflict of interest declaration[A]**	**Overall confidence[E]**
Sella, 2024 [29]	Yes	Partial Yes	Yes	Yes	Yes	Yes	No	Partial Yes	Partial Yes	No	Yes	Yes	No	Yes	No	Yes	Critically Low
Zhang, 2023 [30]	Yes	Partial Yes	Yes	No	Yes	Yes	No	Partial Yes	Partial Yes	No	No	No	No	Yes	No	Yes	Critically Low
Valachis, 2015 [31]	Yes	No	No	Partial Yes	No	Yes	No	No	No	No	No	No	No	Yes	Yes	Yes	Critically Low
Zampieri, 2015 [32]	Yes	Yes	No	Partial Yes	Yes	Yes	No	Partial Yes	Partial Yes	No	No	No	No	Yes	No	Yes	Critically Low
Liu, 2015 [33]	Yes	No	No	Partial Yes	Yes	No	No	No	Partial Yes	No	Yes	No	No	Yes	Yes	Yes	Critically Low
Russel, 2016 [38]	Yes	Partial Yes	Yes	No	Yes	No	No	Partial Yes	Partial Yes	No	No MA	No MA	Yes	No	No	Yes	Critically Low
Sole-Lleonart, 2017 [34]	Yes	Partial Yes	No	Yes	Yes	Yes	No	Partial Yes	Partial Yes	No	Yes	No	No	Yes	No	Yes	Critically Low
Qin, 2021 [35]	Yes	Yes	Yes	Partial Yes	No	Yes	No	Partial Yes	Partial Yes	No	Yes	No	No	Yes	Yes	Yes	Critically Low
Vardakas, 2018 [36]	Yes	No	Yes	Partial Yes	No	Yes	No	Partial Yes	Partial Yes	No	Yes	Yes	Yes	Yes	Yes	Yes	Critically Low
Xu, 2018 [37]	Yes	No	No	Partial Yes	No	No	No	Partial Yes	Partial Yes	No	Yes	No	No	Yes	Yes	Yes	Critically Low

AMSTAR 2, A MeaSurement Tool to Assess Systematic Reviews 2 (Shea et al. 2017; doi: 10.1136/bmj.j4008); PICO, participant, intervention, comparison, outcome; RoB, Risk of bias. *Asterisk indicates a critical item (domain). APossible responses: Yes/No. BPossible responses: Yes/Partial Yes/No. A 'Partial Yes' response is evaluated positively to the overall confidence rating. CItem response depends on separate assessment of randomized controlled trials (RCTs) and non-randomized studies of healthcare interventions (NRSI) in the review. DPossible responses: Yes/No/No MA. A 'No MA' response stands for 'no meta-analysis conducted' and does not affect the overall confidence rating. ECritically Low/Low/Moderate/High confidence in the results of the review. Critically Low: more than one critical flaw with or without non-critical weaknesses, Low: One critical flaw with or without non-critical weaknesses, Moderate: No critical flaws but more than one non-critical weakness, High: No flaws at all or one non-critical weakness.

Search Strategy

PubMed

"Nebulizers and Vaporizers", "Administrations, Inhalation", "Pneumonia, Ventilator-Associated", "Anti-Bacterial Agents", "Outcome", and "Drug-Related Side Effects and Adverse Reactions". Other search keywords were: "aerosolized", "nebulized", "inhaled", "antibiotics", "antimicrobials", "ventilator-associated tracheobronchitis", "ventilator-associated pneumonia", "pulmonary infections", "lung infections", "respiratory tract infections", "hospital-acquired pneumonia", "bacterial eradication", "clinical cure", "microbiological cure", "mortality", "length of stay", and "side effects".

#1"Aerosols" [Mesh]
#2 Administrations, Inhalation [mesh]
#3"Nebulizers and Vaporizers" [mesh]
#4 nebuliz*
#5 aerosol*
#6 inhal*
#1 OR #2 OR #3 OR #4 OR #5 OR #6
AND
#7"Anti-bacterial agents"
#8"Antibiotics"
#9 "Antimicrobials"
#7 OR #8 OR #9
AND
#10 "Pneumonia, Ventilator-Associated" [mesh]
#11 "Ventilator-associated pneumonia*
#12 "Ventilator-associated tracheobronchitis"
#13 "Hospital-acquired pneumonia*
#14 "Pulmonary infections"
#15 "Lung infections"
#16 "Respiratory tract infections"
#17 "Outcome" [mesh]
#18 "Drug-Related Side Effects and Adverse Reactions" [mesh]
#19 "Side effects"
#20 "Bacterial eradication"
#21 "Microbiological cure"
#22 "Clinical cure"
#23 "Mortality"
#24 "Length of stay"

#10 OR #11 OR #12 OR #13 OR #14 OR #15 OR #16 OR #17 OR #18 OR #19 OR #20 OR #21 OR #22 OR #23 OR #24

Epistemonikos

Search string

nebulized OR aerosolized AND antibiotic AND pneumonia

Cochrane Library of Systematic Reviews

Search string

(vap OR pneumonia) and (nebulised or aerosolized) AND (antibiotics OR antimicrobial)

PROSPERO

Search string

(aerolized or nebulized) AND (antibiotic or antimicrobial or colistin or amikacin or gentamycin or tobramycin) AND vap

R code

a. Overlap evaluation and visualizations

```
##### overlap evaluation, by outcome
#without considering structural missingness
#dataset marios overview
#intalling ccaR
library(devtools)
devtools::install_github('thdiakon/ccaR')
library(ccaR)

#corrected covered area index for CLINICAL RESPONSE
#sheet "clinical response"
cca(marios_overview)
tb1 <- cca_table(marios_overview)
cca_heatmap(marios_overview)
#RESULT: 8 reviews, cca_percentage 18.3%

#corrected covered area index for MICROBIOLOGICAL ERADICATION
#sheet “microbiological eradication”
cca(marios_overview)
#RESULT: 6 reviews, cca_percentage 18.5%

#corrected covered area index for MORTALITY
# sheet "mortality"
cca(marios_overview)
#RESULT: 8 reviews, cca_percentage 18.4%

#corrected covered area index for PNEUMONIA-ASSOCIATED MORTALITY
#sheet "pneumonia-associated mortality"
cca(marios_overview)
tb_pn <- cca_table(marios_overview)
#RESULT: 3 reviews, cca_percentage 9.1%

#corrected covered area index for NEPHROTOXICITY
#sheet "nephrotoxicity"
cca(marios_overview)
#RESULT: 6 reviews, cca_percentage 25%

#corrected covered area index for BRONCHOSPASM
#sheet “bronchospasm”
cca(marios_overview)
#RESULT: 2 reviews, cca_percentage 0%
```

```
#corrected covered area index for MECHANICAL VENTILATION DURATION
#sheet “mechanical ventilation”
cca(marios_overview)
#RESULT: 3 reviews, cca_percentage 10%

#corrected covered area index for ICU length of stay
#sheet “ICU stay”
cca(marios_overview)
 ##RESULT: 2 reviews, cca_percentage 0%
```

b. AMSTAR-2 graphics

```
#####AMSTAR-2 visualizations
#necessary add-ons
install.packages(c("devtools", "tidyverse",
                   "gridExtra", "gt"))
#install anstar2Vis
devtools::install_github('bougioukas/amstar2Vis')
library(amstar2Vis)
#import the dataset
library(readxl)
amstar <- read_excel("Documents/amstar.xlsx")
View(amstar)
tb <- amstar2_table(amstar)
View(tb)
amstar2_gtable(amstar)
amstar2_gtable(amstar) |>
  gt::gtsave("my_table.docx")
getwd()

#gt table
amstar2_gtable(amstar) |>
  gt::gtsave("my_table.docx", path = "C:/path/to/
Users/macbookpro")

##visualizations
#barplot
amstar2_barplot(amstar)
#overall confidence plot
amstar2_overall(amstar)
```

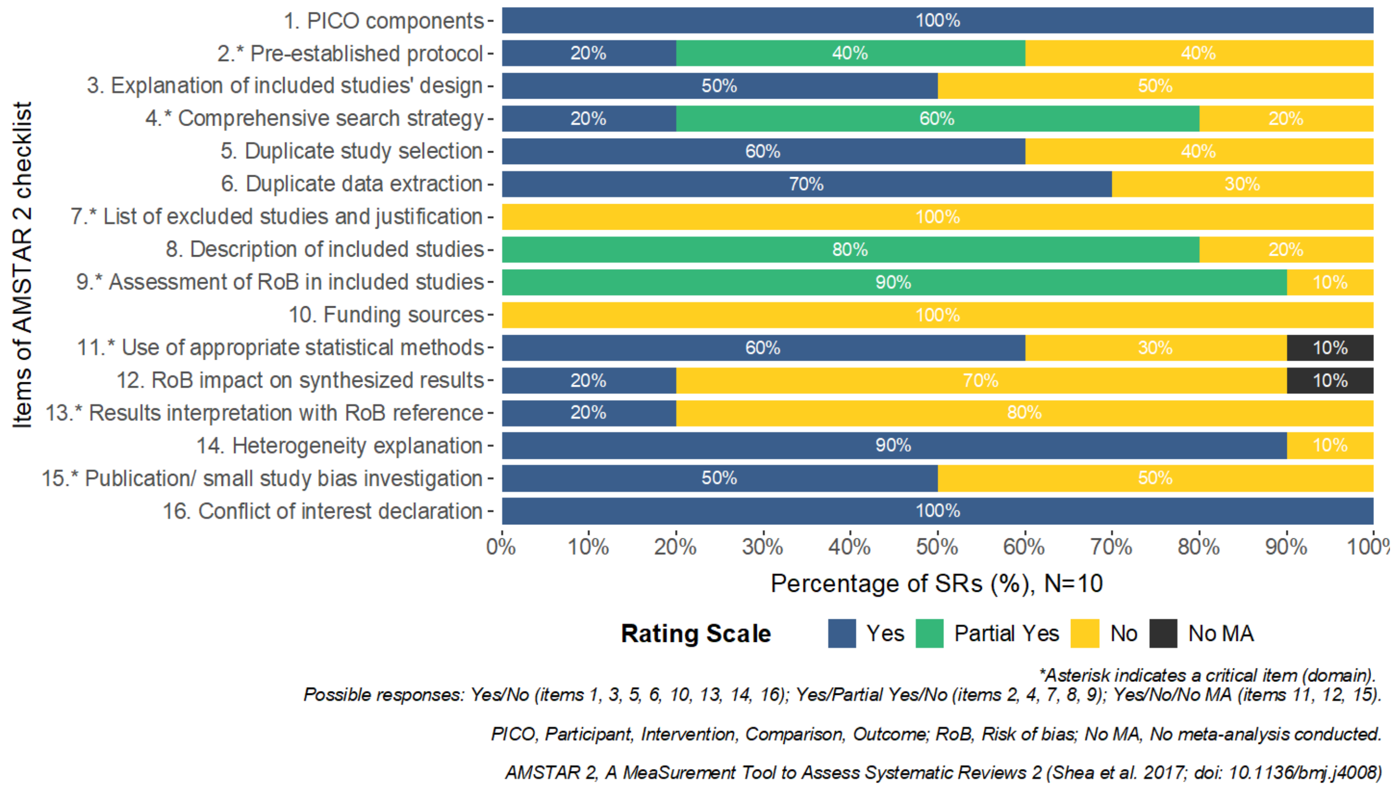

Figure S1. AMSTAR 2 barplot.

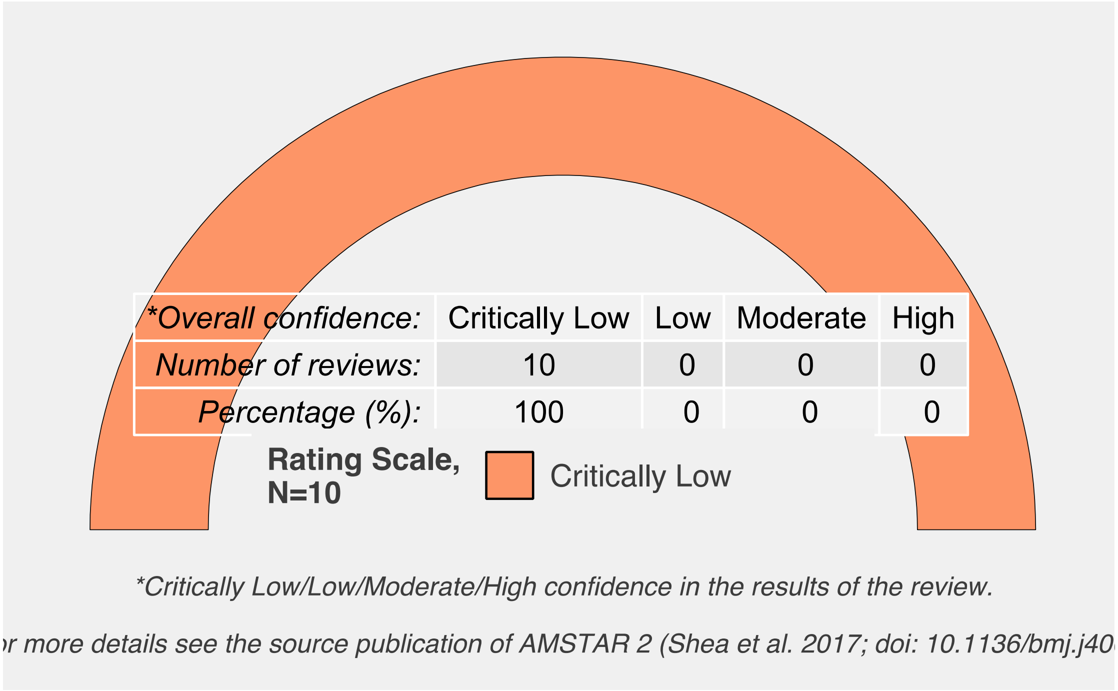

Figure S2. AMSTAR 2 overall confidence.

Heatmaps displaying corrected cover area indices

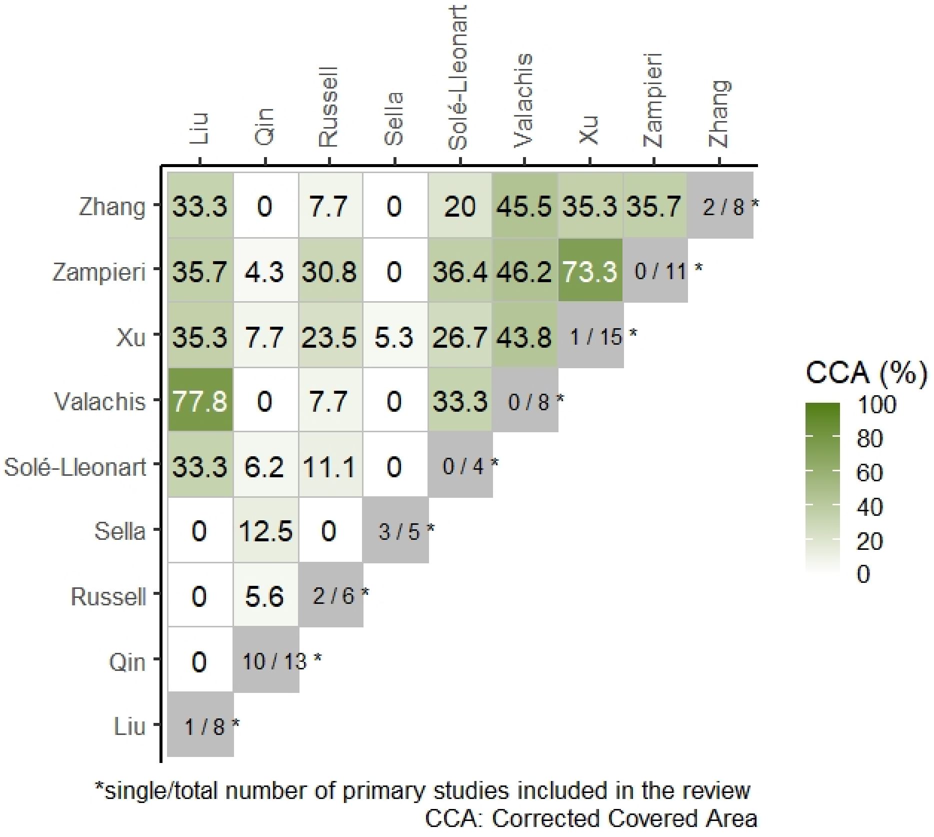

Figure S3. Clinical response. CCA percentage 15.4%.

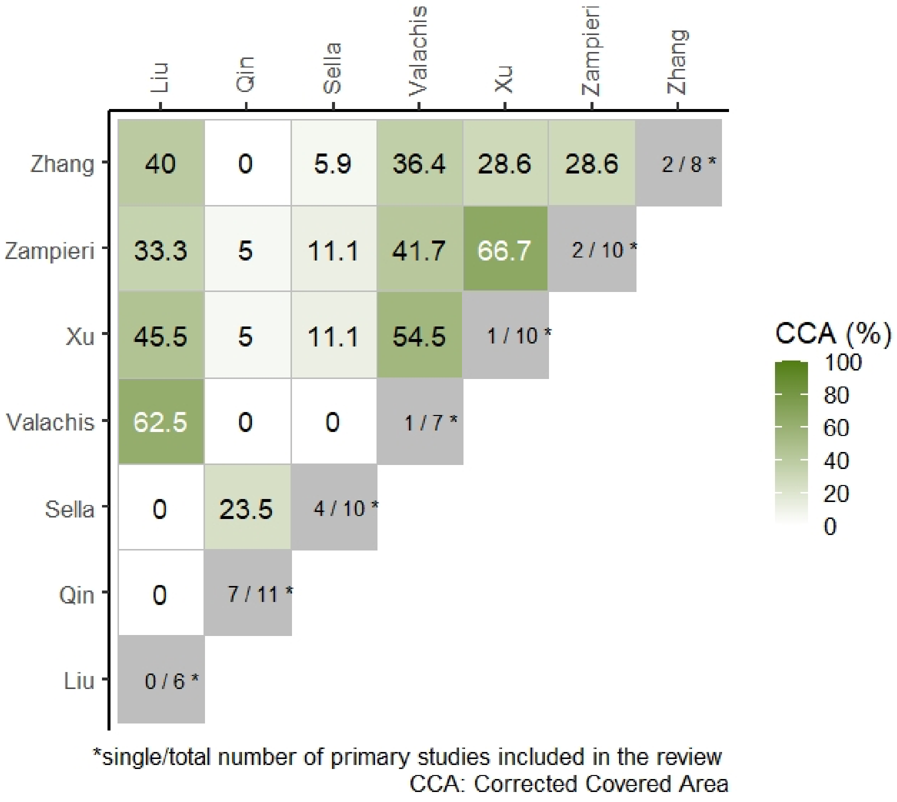

Figure S4. Microbiological eradication. CCA percentage 16.7%.

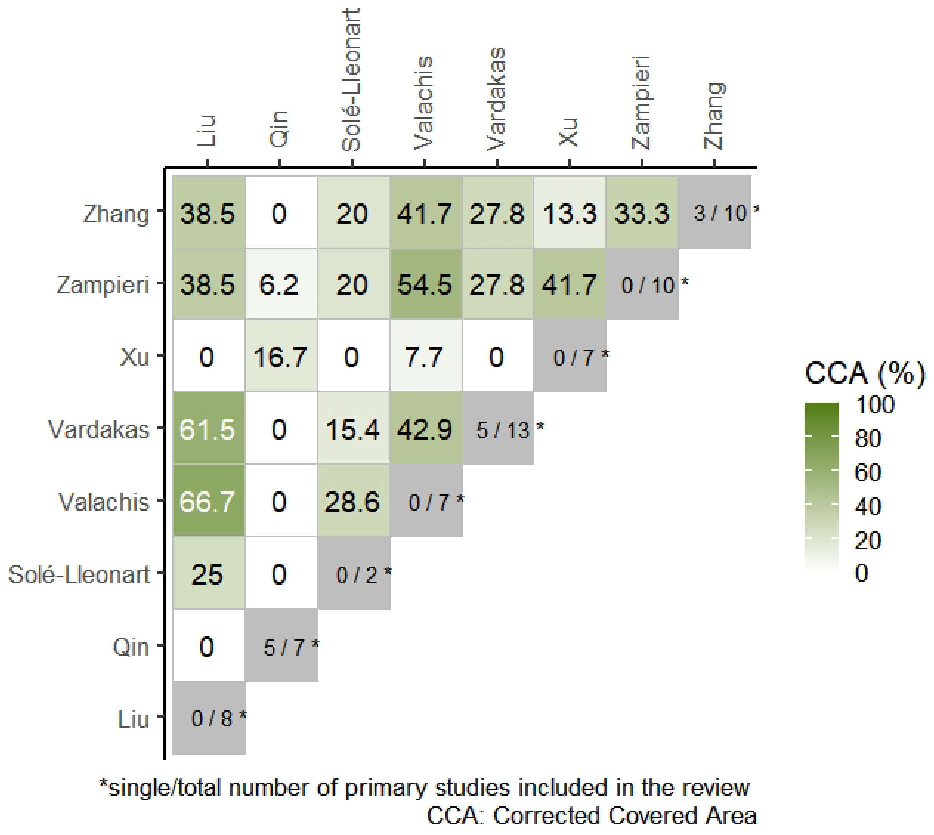

Figure S5 All-cause mortality. CCA percentage 18.4%.

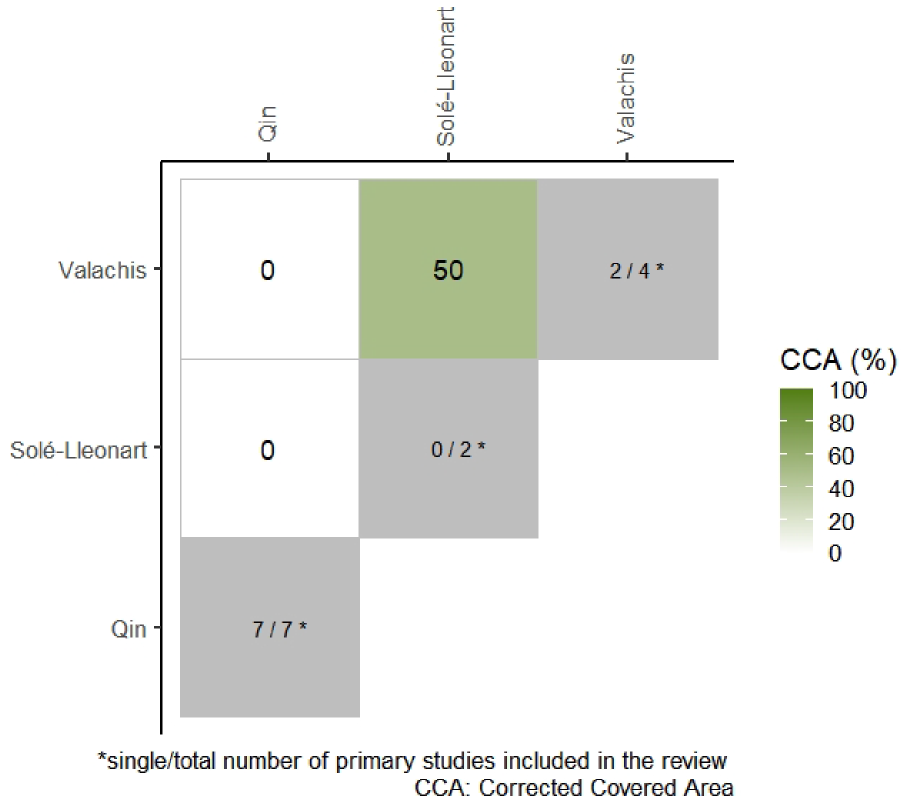

Figure S6. Pneumonia-associated mortality. CCA percentage 9.1%.

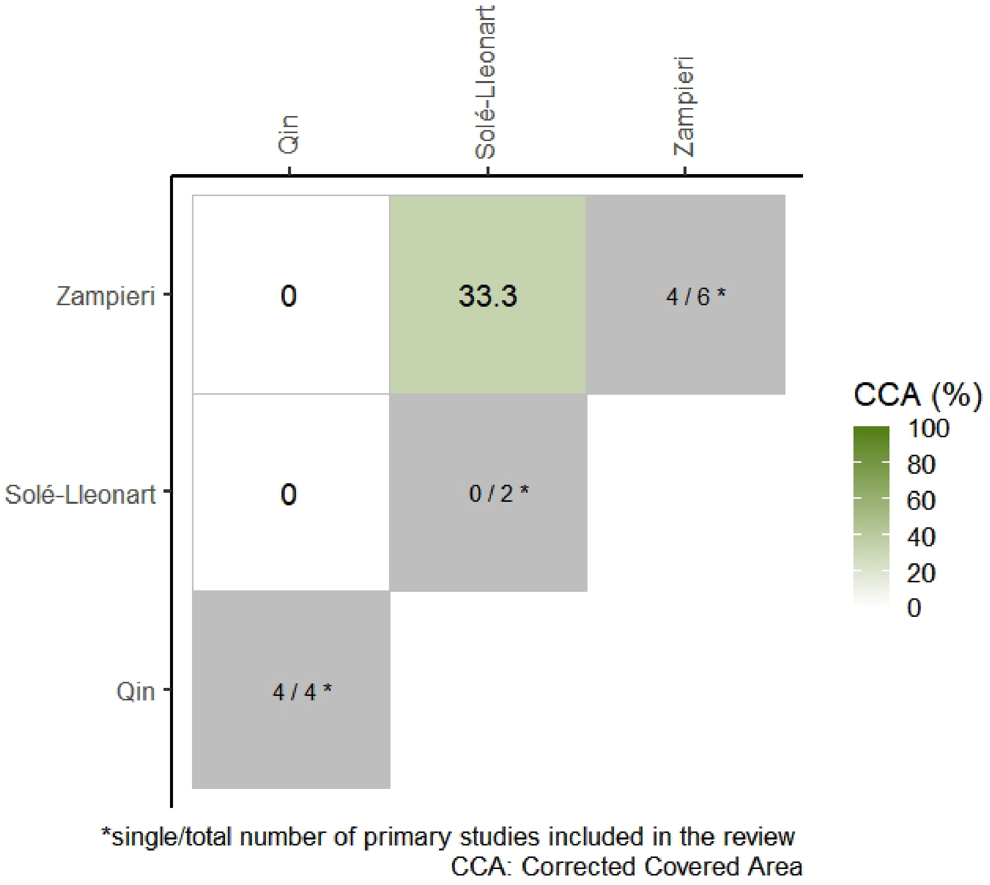

Figure S7. Duration of mechanical ventilation. CCA percentage 10%.

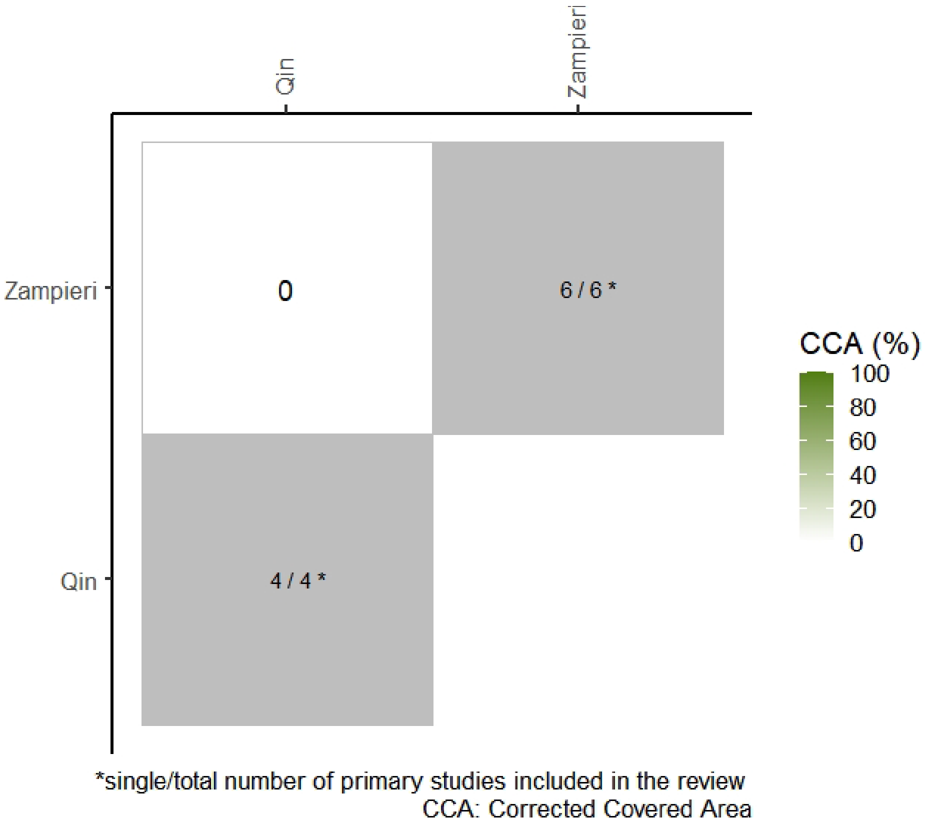

Figure S8. ICU length of stay. CCA percentage 0%.

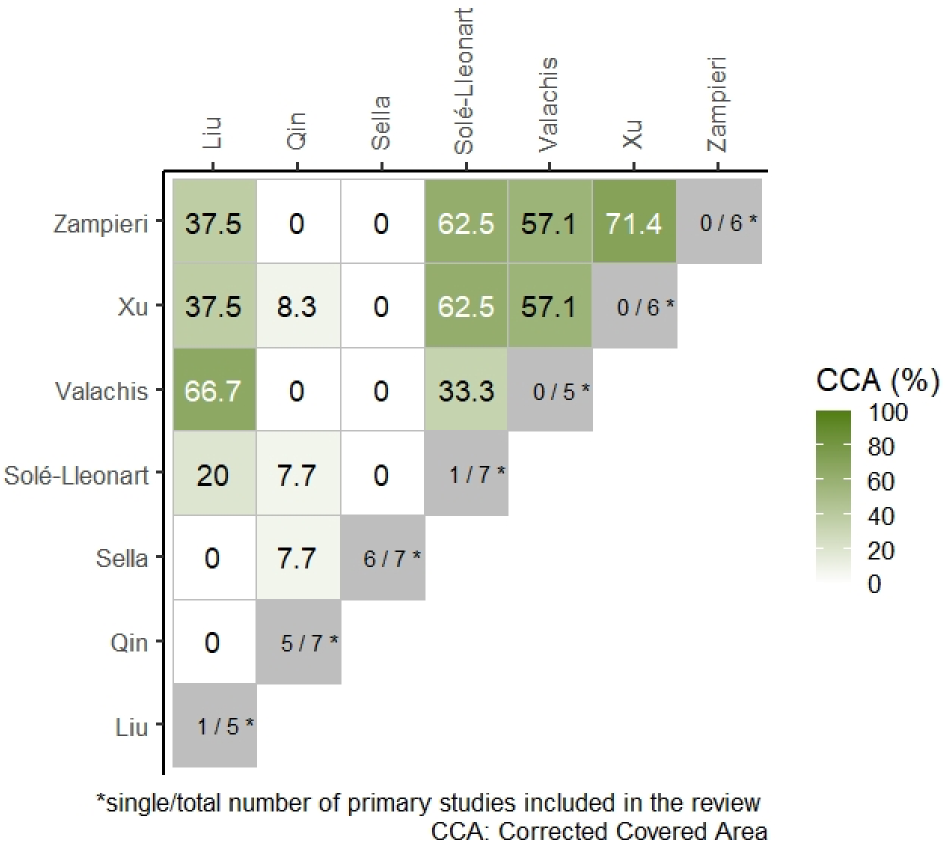

Figure S9. Nephrotoxicity. CCA percentage 15.9%.

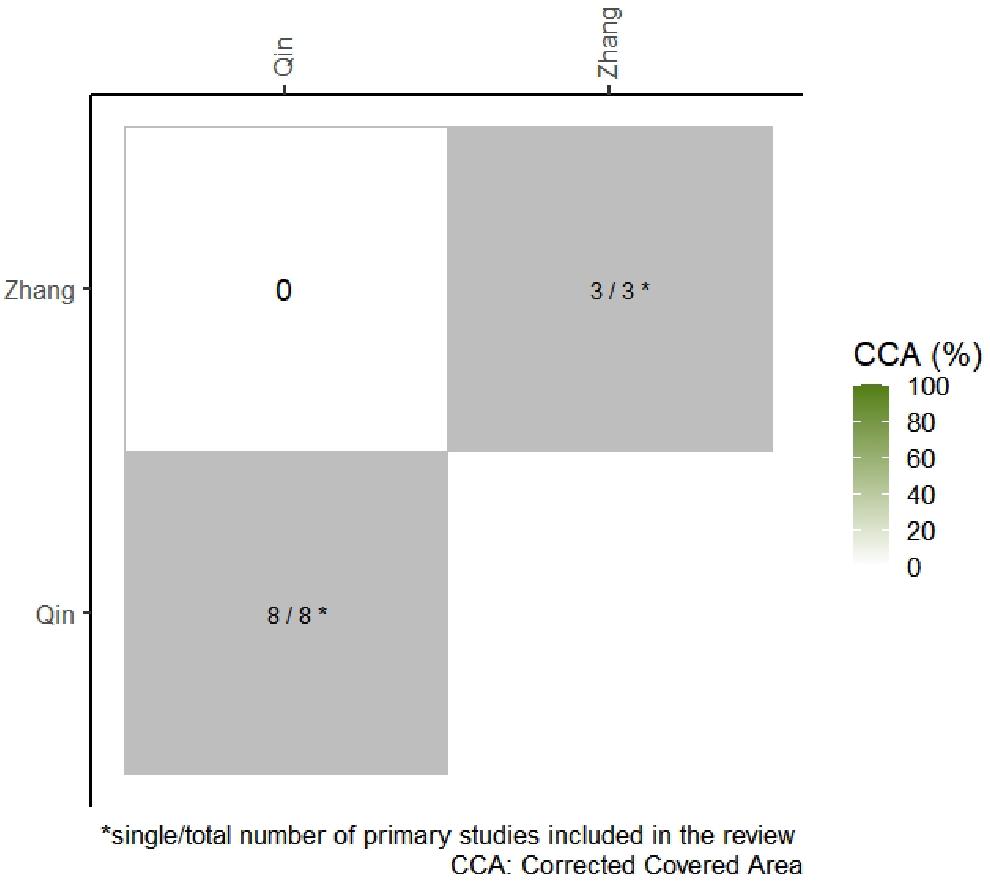

Figure S10. Bronchospasm. CCA percentage 0%.

PRIOR Checklist

(Gates M, Gates A, Pieper D, et al. Reporting guideline for overviews of reviews of healthcare interventions: development of the PRIOR statement. *BMJ* 2022; 378: e070849. doi:10.1136/bmj-2022-070849.)

**Section**<br>Topic	**#**	**Item**	**Location reported**
**TITLE**			
Title	1	Identify the report as an overview of reviews.	Title
**ABSTRACT**			
Abstract	2	Provide a comprehensive and accurate summary of the purpose, methods, and results of the overview of reviews.	Page 1
**INTRODUCTION**			
Rationale	3	Describe the rationale for conducting the overview of reviews in the context of existing knowledge.	Page 3, §1
Objectives	4	Provide an explicit statement of the objective(s) or question(s) addressed by the overview of reviews.	Page 3, §3
**METHODS**			
Eligibility criteria	5a	Specify the inclusion and exclusion criteria for the overview of reviews. If supplemental primary studies were included, this should be stated, with a rationale.	Page 11, inclusion criteria
	5b	Specify the definition of 'systematic review' as used in the inclusion criteria for the overview of reviews.	Page 11, two-group comparison, efficacy and safety outcomes
Information sources	6	Specify all databases, registers, websites, organizations, reference lists, and other sources searched or consulted to identify systematic reviews and supplemental primary studies (if included).<br>Specify the date when each source was last searched or consulted.	Page 11, information sources subsection
Search strategy	7	Present the full search strategies for all databases, registers and websites, such that they could be reproduced. Describe any search filters and limits applied.	Supplementary material
Selection process	8a	Describe the methods used to decide whether a systematic review or supplemental primary study (if included) met the inclusion criteria of the overview of reviews.	Page 11, inclusion and exclusion criteria
	8b	Describe how overlap in the populations, interventions, comparators, and/or outcomes of systematic reviews was identified and managed during study selection.	Pages 11, 12, study selection subsection
Data collection process	9a	Describe the methods used to collect data from reports.	Page 12, data extraction subsection
	9b	If applicable, describe the methods used to identify and manage primary study overlap at the level<br>of the comparison and outcome during data collection. For each outcome, specify the method used to illustrate and/or quantify the degree of primary study overlap across systematic reviews.	Page 12, methodological quality subsection
	9c	If applicable, specify the methods used to manage discrepant data across systematic reviews during data collection.	Not applicable
Data items	10	List and define all variables and outcomes for which data were sought. Describe any assumptions made and/or measures taken to identify and clarify missing or unclear	Page 12, data extraction subsection

		information.	
Risk of bias assessment	11a	Describe the methods used to *assess* risk of bias or methodological quality of the included systematic reviews.	Page 12, methodological quality subsection
	11b	Describe the methods used to *collect* data on (from the systematic reviews) and/or *assess* the risk of bias of the primary studies included in the systematic reviews. Provide a justification for instances where flawed, incomplete, or missing assessments are identified but not re-assessed.	Not applicable
	11c	Describe the methods used to *assess* the risk of bias of supplemental primary studies (if included).	Page 12, methodological quality and risk of bias subsection
Synthesis methods	12a	Describe the methods used to summarize or synthesize results and provide a rationale for the choice(s).	Not applicable
	12b	Describe any methods used to explore possible causes of heterogeneity among results.	Not applicable
	12c	Describe any sensitivity analyses conducted to assess the robustness of the synthesized results.	Not applicable
Reporting bias assessment	13	Describe the methods used to *collect* data on (from the systematic reviews) and/or *assess* the risk of bias due to missing results in a summary or synthesis (arising from reporting biases at the levels of the systematic reviews, primary studies, and supplemental primary studies, if included).	Not applicable
Certainty assessment	14	Describe the methods used to *collect* data on (from the systematic reviews) and/or *assess* certainty (or confidence) in the body of evidence for an outcome.	Page 12, certainty assessment subsection
**RESULTS**			
	15a	Describe the results of the search and selection process, including the number of records screened, assessed for eligibility, and included in the overview of reviews, ideally with a flow diagram.	Figure 1
	15b	Provide a list of studies that might appear to meet the inclusion criteria, but were excluded, with the main reason for exclusion.	Table S1

**Section** Topic	#	**Item**	**Location reported**
Characteristics of systematic reviews and supplemental primary studies	16	Cite each included systematic review and supplemental primary study (if included) and present its characteristics.	Table 1
Primary study overlaps	17	Describe the extent of primary study overlap across the included systematic reviews.	Outcomes, Table 3, Supplement
Risk of bias in systematic reviews, primary studies, and supplemental primary studies	18a	Present assessments of risk of bias or methodological quality for each included systematic review.	Table 1 and Supplement, AMSTAR table and graphs
	18b	Present assessments (*collected* from systematic reviews or *assessed*	Table 2

		anew) of the risk of bias of the primary studies included in the systematic reviews.	
	18c	Present assessments of the risk of bias of supplemental primary studies (if included).	Results, prevention assessment
Summary or synthesis of results	19a	For all outcomes, summarize the evidence from the systematic reviews and supplemental primary studies (if included). If meta-analyses were done, present for each the summary estimate and its precision and measures of statistical heterogeneity. If comparing groups, describe the direction of the effect.	Table 2
	19b	If meta-analyses were done, present results of all investigations of possible causes of heterogeneity.	Not applicable
	19c	If meta-analyses were done, present results of all sensitivity analyses conducted to assess the robustness of synthesized results.	Not applicable
Reporting biases	20	Present assessments (*collected* from systematic reviews and/or *assessed* anew) of the risk of bias due to missing primary studies, analyses, or results in a summary or synthesis (arising from reporting biases at the levels of the systematic reviews, primary studies, and supplemental primary studies, if included) for each summary or synthesis assessed.	Table 4
Certainty of evidence	21	Present assessments (*collected* or *assessed* anew) of certainty (or confidence) in the body of evidence for each outcome.	Table 4, Page 8
**DISCUSSION**			
Discussion	22a	Summarize the main findings, including any discrepancies in findings across the included systematic reviews and supplemental primary studies (if included).	Page 9, discussion's first paragraph
	22b	Provide a general interpretation of the results in the context of other evidence.	Page 9, first and second paragraphs
	22c	Discuss any limitations of the evidence from systematic reviews, their primary studies, and supplemental primary studies (if included) included in the overview of reviews. Discuss any limitations of the overview of reviews methods used.	Page 12, last paragraph
	22d	Discuss implications for practice, policy, and future research (both systematic reviews and primary research). Consider the relevance of the findings to the end users of the overview of reviews, e.g., healthcare providers, policymakers, patients, among others.	Page 10, second paragraph
**OTHER INFORMATION**			
Registration and protocol	23a	Provide registration information for the overview of reviews, including register name and registration number, or state that the overview of reviews was not registered.	Page 10, methods, last paragraph
	23b	Indicate where the overview of reviews protocol can be accessed, or state that a protocol was not prepared.	Page 10, methods, first paragraph

	23c	Describe and explain any amendments to information provided at registration or in the protocol. Indicate the stage of the overview of reviews at which amendments were made.	Minor amendments in the initial stages of the review. Reviewer was added
Support	24	Describe sources of financial or non-financial support for the overview of reviews, and the role of the funders or sponsors in the overview of reviews.	No support was granted
Competing interests	25	Declare any competing interests of the overview of reviews' authors.	None to declare
Author information	26a	Provide contact information for the corresponding author.	Page 1
	26b	Describe the contributions of individual authors and identify the guarantor of the overview of reviews.	Page 13
Availability of data and other materials	27	Report which of the following are available, where they can be found, and under which conditions they may be accessed: template data collection forms; data collected from included systematic reviews and supplemental primary studies; analytic code; any other materials used in the overview of reviews.	Supplementary material
